# 214. Clinical Characteristics and Outcomes of Patients Treated with Tecovirimat for Mpox Disease: A Large Cohort in New York City

**DOI:** 10.1093/ofid/ofae631.072

**Published:** 2025-01-29

**Authors:** Ciarra Leocadio, Elizabeth A Garcia, Maura Lash, Jonathan Berardi, Justin Chan, Rachel Chasan, Blanca C Esquivel, Mary K Foote, Marshall J Glesby, Amiyah Guerra, Alexander B Harris, Dorothy Knutsen, Sharon Mannheimer, Dana Mazo, Jacob McLean, Tristan D McPherson, Eric Meyerowitz, Ofole Mgbako, Gopi Patel, Patricia Pagán Pirallo, Asa Radix, Paul F Riska, Mark N Sayegh, Raphael Shaw, Barry S Zingman, Rustin A Zomorodi, Jason Zucker, Marcia Wong

**Affiliations:** New York City Department of Health, Queens, New York; New York City Department of Health and Mental Hygiene, Brooklyn, New York; NYC DOHMH, New York, New York; Weill Cornell Medical College, New York, New York; NYU Grossman School of Medicine, New York City, New York; The Mount Sinai Hospital, New York, New York; Callen-Lorde Community Health Center, New York, New York; New York City Department of Health and Mental Hygiene, Brooklyn, New York; Weill Cornell Medicine, New York, New York; Callen Lorde Community Health Center, Manhattan, New York; Callen-Lorde Community Health Center, New York, New York; NYU Langone, New York, New York; Harlem Hospital Center/Columbia University, New York, New York; New York University, New York, NY; New York Presbyterian - Columbia University Irving Medical Center, New York, New York; New York City Department of Health and Mental Hygiene, Brooklyn, New York; Montefiore Medical Center / Albert Einstein College of Medicine, Bronx, New York; NYU Grossman School of Medicine, New York City, New York; Icahn School of Medicine at Mount Sinai, New York, NY; NYU Grossman School of Medicine, New York City, New York; Callen-Lorde Community Health Center, New York, New York; Montefiore Medical Center, Bronx, New York; NYC Health + Hospitals/ Harlem Columbia University, scarsdale, New York; Waterbury Hospital , New Rochelle , New York; Montefiore Medical Center and Albert Einstein College of Medicine, Bronx, NY; Icahn School of Medicine at Mount Sinai, New York, NY; Columbia University Irving Medical Center, New York, NY; New York City Department of Health and Mental Hygiene, Brooklyn, New York

## Abstract

**Background:**

Clinical characteristics and outcomes in patients with mpox treated with tecovirimat are not well described.

**Figure:**
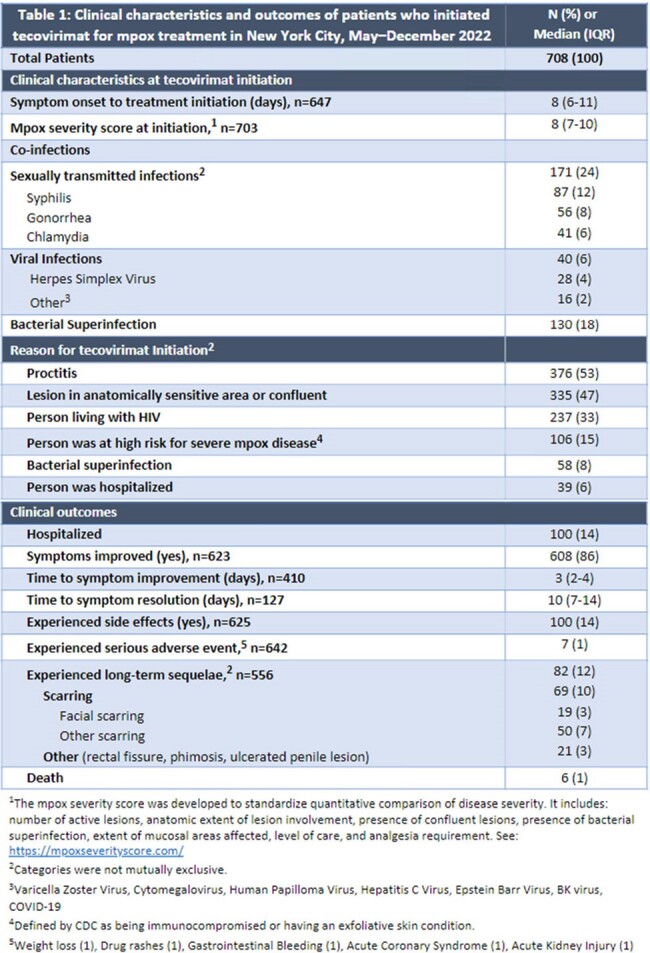

**Methods:**

We requested retrospective deidentified data from healthcare systems in New York City (NYC) with >50 tecovirimat prescriptions. Patients with probable or confirmed mpox initiating tecovirimat from May–December 2022 were included. We used a survey tool to extract demographic and clinical data from medical records. Patients reporting tecovirimat initiation at a different institution or with unknown HIV status were excluded. We used multivariable Poisson regression models with robust standard errors to examine factors associated with hospitalization and treatment delays.

**Figure:**
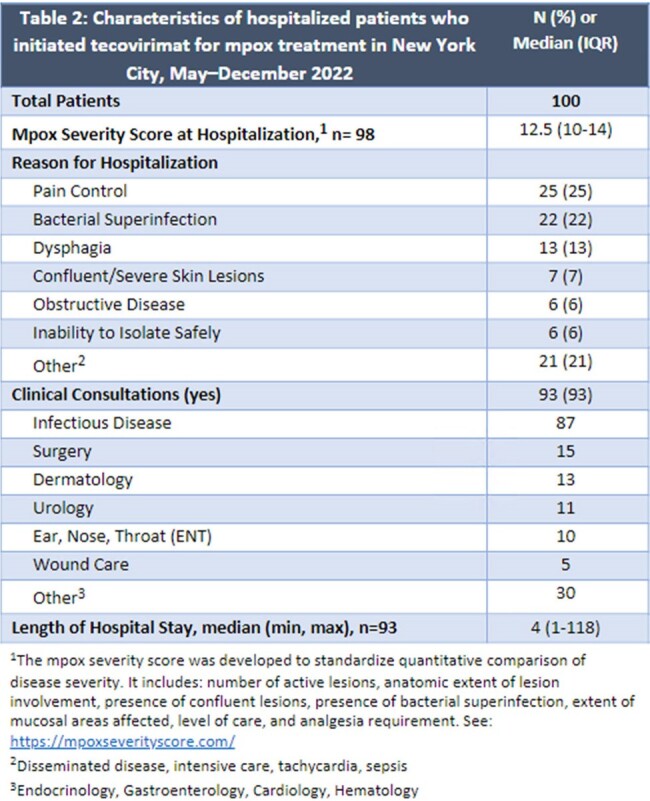

**Results:**

Of 751 eligible records, we excluded 41, leaving 708 patients in the analysis; median age was 36 years (IQR 31–43); 694 (98%) were assigned male at birth; 566 (80%) were cisgender men who have sex with men; 399 (56%) were living with HIV; and 232 (33%), 195 (28%), and 182 (26%) identified as Hispanic, non-Hispanic (NH) white, and NH Black, respectively. Reasons for treatment included proctitis (n=376, 53%), lesion type (confluent, location) (335, 47%), HIV (237, 33%), and bacterial superinfection (58, 8%). Side effects (100, 14%) and severe adverse events (7, 1%) were rare. The most common long-term sequela was scarring (69,10%) (Table 1). Of 100 (14%) hospitalized patients, reasons for hospitalization included pain (25%), bacterial superinfection (22%), and dysphagia (13%) (Table 2). Median delay from symptom onset to treatment initiation was 8 days (IQR 6–11). NH Black patients had higher risk of initiating ≥8 days after symptom onset (adjusted relative risk [aRR]=1.4, 95%CI: 1.2–1.7) and being hospitalized (aRR=2.2, 95%CI: 1.4–3.5) compared with Hispanic patients, adjusting for HIV and insurance status (Tables 3,4).

**Figure:**
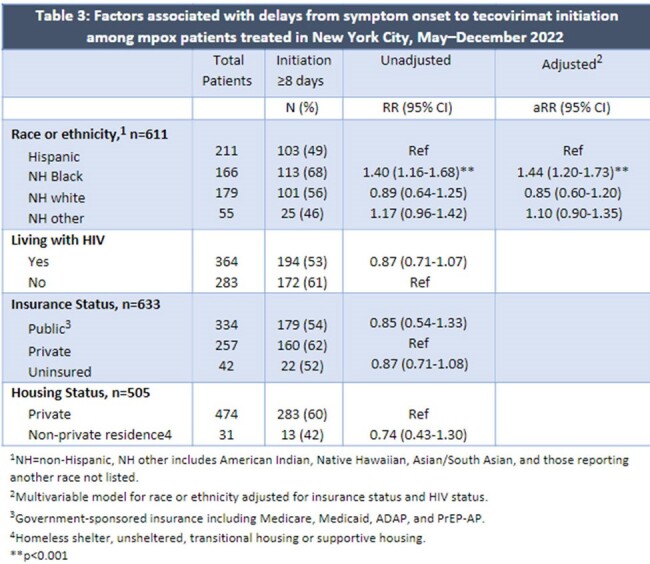

**Conclusion:**

We described common reasons for tecovirimat initiation and hospitalization. There were racial and ethnic disparities in hospitalization and treatment delays that may have been due to late presentation to care or provider-related delayed testing. More research and focused efforts to mitigate these disparities are needed. These findings may better inform decisions for treatment initiation and hospitalization for mpox.

**Figure:**
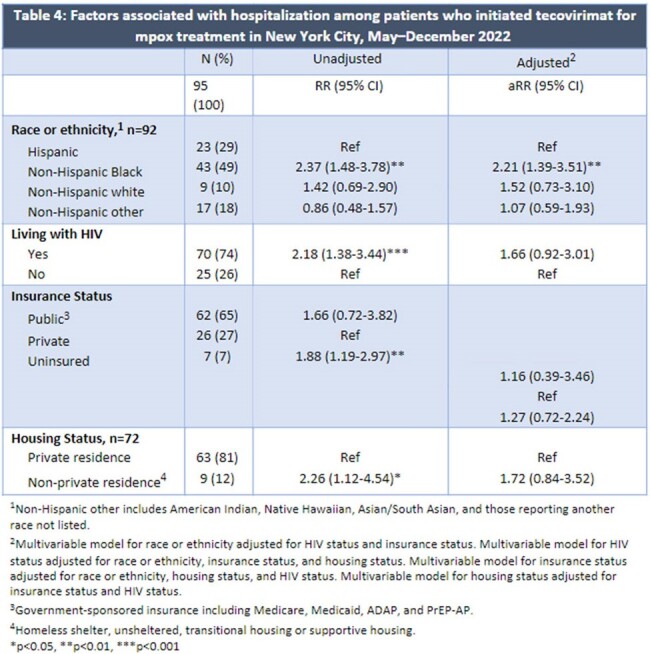

**Disclosures:**

**Ofole Mgbako, MD MS**, Gilead: Advisor/Consultant

